# Early warning scores for predicting in-hospital mortality in patients with acute aortic dissection

**DOI:** 10.3389/fmed.2026.1895620

**Published:** 2026-07-13

**Authors:** Mao Hu, Nan Xie, Lvna Xiang

**Affiliations:** 1 Emergency Department of West China Hospital, Sichuan University/West China School of Nursing, Sichuan University, Chengdu, China; 2 Institute of Disaster Medicine, Sichuan University, Chengdu, China; 3 Nursing Key Laboratory of Sichuan Province, Chengdu, China; 4 Department of Outpatient, West China Hospital, Sichuan University, Chengdu, China

**Keywords:** acute aortic dissection, early warning scores, in-hospital mortality, national early warning score, prognosis

## Abstract

**Objective:**

To evaluate the prognostic value of early warning score (EWS) in predicting in-hospital mortality among patients with acute aortic dissection (AAD).

**Methods:**

We retrospectively analyzed consecutive patients diagnosed with AAD who presented to the emergency department between January 1 and December 31, 2022. Collected data included demographic information, clinical characteristics, EWS, laboratory findings, and time to computed tomography angiography (CTA). Univariable and multivariable logistic regression analyses were performed to identify risk factors for in-hospital mortality. Subgroup analyses were conducted according to dissection type (A vs. B).

**Results:**

A total of 510 AAD patients were included, with an in-hospital mortality rate of 33.3%. Univariable analysis revealed that older age, Type A AAD, male sex, elevated EWS, higher lactate, elevated BUN, lower bicarbonate, and shorter time to CTA were significantly associated with mortality (all *p* < 0.05). Multivariable analysis identified Type A AAD and surgical intervention as independent predictors of mortality. Subgroup analysis showed that among patients with Type A AAD, age, surgical treatment, and shorter time to CTA remained independently associated with in-hospital mortality. NEWS demonstrated the highest discriminative performance among the three EWS (AUC = 0.581, 95% CI 0.528–0.634), although its overall predictive ability was modest.

**Conclusion:**

NEWS demonstrated the highest discriminative performance among the evaluated EWS and may serve as a useful tool for initial risk stratification in patients with AAD. Type A AAD and surgical treatment were the only independent predictors of in-hospital mortality, highlighting the importance of timely surgical intervention for improving patient outcomes.

## 1 Introduction

Acute Aortic Dissection (AAD) is an acute and life-threatening cardiovascular condition characterized by a tear in the inner layer of the aortic wall, allowing blood to enter the middle layer and creating a false lumen (1–4). This can lead to a series of severe complications, such as aortic rupture, cardiac tamponade, and acute organ ischemia (5, 6). Although the incidence of AAD is relatively low, it progresses rapidly and has a poor prognosis (7). Without treatment, the mortality rate within 24 h can reach 20–30%, and within a week, it can rise to 50% or even higher (8).

Emergency physicians often rely on clinical scoring systems and imaging studies to assist in diagnosis and decision-making (9). However, given the urgency and limited resources, accurately and quickly assessing the severity of the patient’s condition and the risk of poor outcomes is essential for optimizing resource allocation and formulating treatment strategies in the emergency department (10). Therefore, finding an effective tool to predict the prognosis of AAD patients, particularly in-hospital mortality, is crucial for improving clinical outcomes.

(EWS) systems are tools based on physiological parameters designed to detect the risk of deterioration by monitoring changes in a patient’s vital signs (11–13). Various EWS systems are currently used in clinical practice, typically incorporating parameters such as blood pressure, heart rate, respiratory rate, body temperature, and consciousness level (13). EWS has been widely employed in the early identification and prognosis of various critical conditions, such as sepsis, acute respiratory distress syndrome, and acute coronary syndrome (14–16). However, studies specifically focusing on AAD patients remain limited, especially within the emergency department, where the effectiveness of EWS as a prognostic tool has not been fully validated. National Early Warning Score (NEWS), Modified Early Warning Score (MEWS) and Standardized Early Warning Score (SEWS) are commonly used early warning systems for identifying patients at risk of clinical deterioration. Although all three scores are based on routinely collected physiological parameters, important differences exist among them. NEWS incorporates oxygen saturation and supplemental oxygen requirements and has been widely adopted for acute care assessment. MEWS is a simpler physiological scoring system primarily based on vital signs and level of consciousness. SEWS was developed as a modified aggregate score for early detection of clinical deterioration and differs slightly in parameter weighting and risk categorization. These differences may influence their prognostic performance in patients with acute aortic dissection. Therefore, the aim of this study is to evaluate and compare the predictive performance of three commonly used EWS (NEWS, MEWS, and SEWS) for in-hospital mortality in patients with AAD and to identify clinical factors independently associated with in-hospital mortality.

## 2 Methods and materials

This study was designed as a retrospective observational study focused on prediction rather than causal inference. By retrospectively analyzing the medical records and relevant data of patients with AAD who presented to the emergency department, the study aims to evaluate the effectiveness of SEWS, NEWS, and MEWS in predicting in-hospital mortality.

### 2.1 Data collection

Of the 705 eligible patients, 195 were excluded because data required for calculation of NEWS, MEWS, or SEWS were incomplete at admission. NEWS, MEWS, and SEWS were calculated using the first recorded vital signs documented at emergency department admission, before any major therapeutic intervention. All patients underwent computed tomography angiography (CTA) confirmation of AAD within 24 h of ED arrival. The data were sourced from the hospital information system (HIS) of a large tertiary hospital’s emergency department, including general patient information and vital signs at the time of emergency admission. Specifically, the data were include demographic information (such as age and sex), Glasgow Coma Scale (GCS) scores, pain scores, and in-hospital clinical outcomes.

Inclusion criteria: Patients who presented to the emergency department and were diagnosed with acute aortic dissection, had complete vital sign records and laboratory test data at admission, and had a confirmed clinical outcome.

This study was approved by the Ethics Committee of West China Hospital of Sichuan University.

### 2.2 Data analysis

Statistical analysis were performed using IBM SPSS Statistics (version 29.0). Continuous variables were presented as mean ± standard deviation (SD) or median with interquartile range (IQR), based on their distribution assessed by the Shapiro–Wilk test. Categorical variables were presented as frequencies and percentages. Between-group comparisons were performed using the independent-samples *t*-test or the Mann–Whitney U test for continuous variables, and the Pearson’s chi-square test or Fisher’s exact test for categorical variables, as appropriate. Univariable logistic regression analysis was performed to identify potential predictors associated with in-hospital mortality. Variables with *p* < 0.10 in univariable analysis or those of clinical importance were included in the multivariable logistic regression model using a stepwise selection method. To avoid potential multicollinearity among EWS derived from similar physiological parameters, only the NEWS, which demonstrated the best discriminatory performance, was included in the final multivariable model.

The discriminative ability of EWS was evaluated using receiver operating characteristic (ROC) curve analysis, and the area under the ROC curve (AUC) was calculated. Pairwise comparisons of AUCs were performed using DeLong’s test. The optimal cutoff value (determined by the Youden index), sensitivity, and specificity were reported. A two-sided *p*-value < 0.05 was considered statistically significant.

## 3 Results

### 3.1 Patient characteristics

A total of 510 patients with AAD were included, with a mean age of 55.24 ± 13.77 years. The majority were male (78.2%, *n* = 399). Overall, 170 patients (33.3%) died during hospitalization, whereas 340 (66.7%) survived. Baseline demographic and clinical characteristics are summarized in Table 1. Compared with survivors, non-survivors were significantly older (60.16 ± 13.62 vs. 52.79 ± 13.20 years, *p* < 0.001), were more likely to have Type A AAD (78.8% vs. 61.2%, *p* < 0.001), and were less likely to be male (71.8% vs. 81.5%, *p* = 0.012). In addition, the proportion of patients undergoing surgical treatment was markedly lower among non-survivors than among survivors (13.5% vs. 78.5%, *p* < 0.001).

**Table 1:** Baseline characteristics of patients diagnosed with AAD.

Clinical variables	Total (*n* = 510)	Survival (*n* = 340)	Death (*n* = 170)	*p*
Type A AAD, n (%)	342 (67.1)	208 (61.2)	134 (78.8)	<0.001
Age (years, mean ± SD)	55.24 ± 13.77	52.79 ± 13.20	60.16 ± 13.62	<0.001
Males, *n*(%)	399 (78.2)	277 (81.5)	122 (71.8)	0.012
Surgical treatment, *n*(%)	290 (56.9)	267 (78.5)	23 (13.5)	<0.001
Glasgow coma scale scores (mean ± SD)	14.75 ± 1.56	14.90 ± 0.96	14.43 ± 2.31	0.011
EWS				
MEWS (mean ± SD)	1.43 ± 0.92	1.34 ± 0.87	1.59 ± 1.00	0.006
NEWS (mean ± SD)	1.82 ± 1.92	1.59 ± 1.68	2.26 ± 2.27	<0.001
SEWS (mean ± SD)	0.71 ± 0.97	0.63 ± 0.89	0.87 ± 1.10	0.008
Pain scores (mean ± SD)	1.45 ± 1.75	1.49 ± 1.75	1.37 ± 1.75	0.475
Lactate (mmol/L, median [IQR])	1.50 (1.20–2.20)	1.40 (1.20–2.10)	1.70 (1.20–2.58)	0.039
Creatinine (μmol/L, median [IQR])	87.50 (71.25–119.00)	81.00 (68.50–111.50)	96.5 (74.00–133.75)	0.002
BUN (mmol/L, median [IQR])	6.05 (4.90–8.95)	6.10 (4.85–8.05)	7.50 (5.15–9.90)	<0.001
Bicarbonate (mmol/L, median [IQR])	21.40 (19.18–22.93)	21.50 (19.95–23.30)	21.60 (17.85–22.3)	0.023
Time to CTA (minutes, median [IQR])	28.00 (13.75–49.00)	29.00 (15.50–54.00)	21.00 (9.50–41.50)	<0.001

AAD, acute aortic dissection; MEWS, Modified Early Warning Score; NEWS, National Early Warning Score; SEWS, Standardized Early Warning Score; BUN, blood urea nitrogen; CTA, computed tomography angiography; SD, standard deviation; IQR, interquartile range.

Among the three EWS, patients who died during hospitalization had significantly higher MEWS (1.59 ± 1.00 vs. 1.34 ± 0.87, *p* = 0.006), NEWS (2.26 ± 2.27 vs. 1.59 ± 1.68, *p* < 0.001), and SEWS (0.87 ± 1.10 vs. 0.63 ± 0.89, *p* = 0.008) than survivors. Furthermore, GCS scores were significantly lower in the mortality group (14.43 ± 2.31 vs. 14.90 ± 0.96, *p* = 0.011). No significant difference in pain scores was observed between the two groups (1.37 ± 1.75 vs. 1.49 ± 1.75, *p* = 0.475).

Laboratory findings indicated higher lactate [1.70 (1.20–2.58) vs. 1.40 (1.20–2.10) mmol/L, *p* = 0.039], creatinine [96.50 (74.00–133.75) vs. 81.00 (68.50–111.50) μmol/L, *p* = 0.002], and blood urea nitrogen (BUN) [7.50 (5.15–9.90) vs. 6.10 (4.85–8.05) mmol/L, *p* < 0.001] in the mortality group, whereas bicarbonate was slightly lower [21.60 (17.85–22.30) vs. 21.50 (19.95–23.30) mmol/L, *p* = 0.023]. Moreover, the median time from emergency department admission to CTA was significantly shorter in patients who died than in those who survived [21.00 (9.50–41.50) vs. 29.00 (15.50–54.00) minutes, *p* < 0.001]. Overall, significant differences between survivors and non-survivors were observed in age, sex, AAD type, surgical treatment, GCS score, all three EWS, lactate, creatinine, BUN, bicarbonate, and time to CTA, whereas pain scores did not differ significantly between the two groups.

### 3.2 Univariable logistic regression analysis

Univariable logistic regression analysis identified several variables significantly associated with in-hospital mortality (Table 2). Increasing age was associated with a higher risk of in-hospital mortality (OR 1.044, 95% CI: 1.028–1.060, *p* < 0.001). Patients with Type A AAD had a significantly increased risk of death compared with those with type B AAD (OR, 2.362, 95% CI, 1.540–3.623, *p* < 0.001). Male sex was associated with a lower risk of in-hospital mortality (OR, 0.578, 95% CI, 0.375–0.890, *p* = 0.013). Higher GCS scores were associated with a reduced risk of death (OR, 0.822, 95% CI, 0.714–0.947, *p* = 0.006).

**Table 2:** Univariable logistic regression analyses of factors associated with in-hospital mortality in patients with AAD.

Clinical variables	OR	95%CI	*p*
Age (years)	1.044	1.028–1.060	<0.001
AAD Type (Type A vs. B)	2.362	1.540–3.623	<0.001
Sex (male vs. female)	0.578	0.375–0.890	0.013
Glasgow Coma Score	0.822	0.714–0.947	0.006
EWS			
MEWS	1.333	1.093–1.624	0.004
NEWS	1.195	1.086–1.315	<0.001
SEWS	1.283	1.065–1.546	0.009
Pain scores	0.962	0.865–1.070	0.474
Creatinine (μmol/L)	1.000	0.999–1.001	0.607
Lactate (mmol/L)	1.324	1.136–1.543	<0.001
BUN (mmol/L)	1.041	1.007–1.076	0.018
Bicarbonate (mmol/L)	0.915	0.866–0.966	0.001
Surgical treatment (Yes vs. No)	0.042	0.025–0.071	<0.001
Time to CTA (minutes)	0.992	0.987–0.998	0.006

OR, odds ratio; CI, confidence interval.

All three EWS were significantly associated with in-hospital mortality. The odds of death increased with higher MEWS (OR 1.333, 95% CI: 1.093–1.624, *p* = 0.004), NEWS (OR 1.195, 95% CI: 1.086–1.315, *p* < 0.001), and SEWS (OR 1.283, 95% CI: 1.065–1.546, *p* = 0.009). Laboratory parameters including lactate (OR 1.324, 95% CI: 1.136–1.543, *p* < 0.001) and BUN (OR 1.041, 95% CI: 1.007–1.076, *p* = 0.018) were associated with an increased risk of mortality, whereas higher bicarbonate levels were associated with a lower risk (OR, 0.915, 95% CI, 0.866–0.966, *p* = 0.001). Patients who underwent surgical treatment had a markedly lower risk of in-hospital mortality than those without surgical treatment (OR, 0.042, 95% CI, 0.025–0.071, *p* < 0.001). Longer time to CTA was associated with a lower odd of in-hospital mortality in the univariable analysis (OR 0.992, 95% CI 0.987–0.998, *p* = 0.006). Pain scores were not significantly associated with in-hospital death (OR 0.962, 95% CI: 0.865–1.070, *p* = 0.474).

Variables with *p* < 0.10 in the univariable analysis, together with clinically relevant variables, were considered candidates for inclusion in the multivariable logistic regression analysis.

### 3.3 Predictive performance of EWS

The discriminative performance of the three EWS for predicting in-hospital mortality was evaluated using ROC analysis (Table 3). Among the three scoring systems, NEWS demonstrated the highest discriminative ability, with an area under the ROC curve (AUC) of 0.581 (95% CI, 0.528–0.634). The corresponding optimal cut-off value yielded a sensitivity of 38.2% and a specificity of 73.2%.

**Table 3:** Receiver operating characteristic (ROC) analysis of EWS for predicting in-hospital mortality in patients with AAD.

EWS	AUC (95% CI)	Sensitivity	Specificity
NEWS	0.581 (0.528–0.634)	0.382	0.732
MEWS	0.566 (0.513–0.619)	0.435	0.662
SEWS	0.560 (0.506–0.613)	0.529	0.568

The AUCs of MEWS and SEWS were 0.566 (95% CI, 0.513–0.619) and 0.560 (95% CI, 0.506–0.613), respectively. Their optimal cut-off values provided sensitivities of 43.5 and 52.9%, with corresponding specificities of 66.2 and 56.8%, respectively.

Although the overall discriminatory performance of all three EWS was modest, NEWS showed the highest predictive performance among the evaluated scoring systems. Given the substantial overlap in physiological variables incorporated into NEWS, MEWS, and SEWS, only NEWS was included in the subsequent multivariable logistic regression analysis to minimize potential multicollinearity.

### 3.4 Multivariable logistic regression

In the multivariable logistic regression analysis, Type A AAD was independently associated with increased odds of in-hospital mortality (OR, 3.146; 95% CI, 1.437–6.887; *p* = 0.004). Patients who underwent surgical treatment had a significantly lower risk of in-hospital mortality compared with those who did not receive Surgical treatment (OR, 0.063; 95% CI, 0.031–0.129; *p* < 0.001). Other variables, including age, sex, NEWS, lactate, BUN, bicarbonate, and time to CTA, were not independently associated with in-hospital mortality (all *p* > 0.05) (Table 4). These findings suggest that Type A AAD and surgical treatment were the only independent predictors of in-hospital mortality in this cohort, whereas the associations observed for age, NEWS, laboratory variables, and time to CTA in the univariable analysis were no longer statistically significant after multivariable adjustment.

**Table 4:** Multivariable logistic regression analyses to detect the prognostic factors for in-hospital mortality in patients with AAD.

Clinical variables	OR	95%CI	*p*
Age (years)	1.026	0.997–1.055	0.076
AAD type (type A vs. B)	3.146	1.437–6.887	0.004
Sex (males vs. female)	1.324	0.570–3.074	0.514
NEWS	1.099	0.915–1.320	0.311
Lactate (mmol/L)	1.150	0.891–1.484	0.282
BUN (mmol/L)	1.021	0.946–1.101	0.595
Bicarbonate (mmol/L)	0.944	0.850–1.049	0.286
Surgical treatment (Yes vs. No)	0.063	0.031–0.129	< 0.001
Time to CTA (minutes)	0.994	0.985–1.002	0.143

OR, odds ratio; CI, confidence interval.

### 3.5 Subgroups analysis by AAD type

Subgroups analyses for type A AAD and type B AAD was performed. The results of univariable analysis showed that in patients with Type A AAD, higher age (OR 1.055, 95% CI: 1.036–1.075), MEWS (OR 1.366, 95% CI: 1.079–1.728), NEWS (OR 1.210, 95% CI: 1.083–1.351), SEWS (OR 1.331, 95% CI: 1.066–1.662), lactate (OR 1.329, 95% CI: 1.113–1.587), and BUN (OR 1.050, 95% CI: 1.005–1.097) were associated with higher in-hospital mortality, while higher bicarbonate level (OR 0.921, 95% CI: 0.865–0.981) and Surgical treatment (OR 0.038, 95% CI: 0.021–0.069) were associated with reduced mortality. A shorter time to CTA was also associated with increased mortality (OR 0.991, 95% CI 0.984–0.999). Among patients with type B AAD, surgical treatment was associated with lower in-hospital mortality in the univariable analysis, whereas no other clinical or laboratory variables reached statistical significance (Table 5).

**Table 5:** Univariable logistic analysis for in-hospital mortality in patients with type A and type B AAD.

Clinical variables	Type A AAD			Type B AAD		
	OR	95%CI	*p*	OR	95%CI	*p*
Age (years)	1.055	1.036–1.075	<0.001	1.010	0.980–1.040	0.528
Sex (males vs. female)	0.649	0.395–1.065	0.087	0.553	0.218–1.400	0.211
Glasgow Coma Scale	0.855	0.742–0.985	0.030	0.153	0.000 –∞	1.000
EWS						
MEWS	1.366	1.079–1.728	0.009	1.144	0.760–1.723	0.518
NEWS	1.210	1.083–1.351	< 0.001	1.026	0.820–1.284	0.823
SEWS	1.331	1.066–1.662	0.012	1.073	0.724–1.591	0.724
Pain scores	0.996	0.878–1.129	0.950	0.920	0.745–1.138	0.443
Creatinine (μmol/L)	1.000	0.999–1.002	0.577	1.001	0.999–1.002	0.417
Lactate (mmol/L)	1.329	1.113–1.587	0.002	1.086	0.671–1.758	0.738
BUN (mmol/L)	1.050	1.005–1.097	0.031	1.030	0.971–1.091	0.327
Bicarbonate (mmol/L)	0.921	0.865–0.981	0.011	0.920	0.821–1.032	0.154
Surgical treatment (Yes vs. No)	0.038	0.021–0.069	<0.001	0.043	0.014–0.132	<0.001
Time to CTA (minutes)	0.991	0.984–0.999	0.020	0.995	0.998–1.002	0.172

OR, odds ratio; CI, confidence interval.

Multivariable logistic regression analysis revealed that age (OR 1.043, 95% CI: 1.008–1.080), Surgical treatment (OR 0.058, 95% CI: 0.025–0.134) and shorter time to CTA (OR 0.986, 95% CI 0.973–1.000, *p* = 0.046) were independently associated with in-hospital mortality among patients with Type A AAD. NEWS, lactate, BUN, bicarbonate, and sex were not independently associated with mortality (Table 6).

**Table 6:** Multivariable logistic regression analyses of predictors of in-hospital mortality in patients with type A AAD.

Clinical variables	OR	95%CI	*p*
Age (years)	1.043	1.008–1.080	0.016
Sex (male vs. female)	0.690	0.254–1.874	0.467
NEWS	1.128	0.906–1.403	0.281
Lactate (mmol/L)	1.198	0.883–1.626	0.245
BUN (mmol/L)	1.002	0.917–1.090	0.967
Bicarbonate (mmol/L)	0.913	0.808–1.032	0.147
Surgical treatment (Yes vs. No)	0.058	0.025–0.134	<0.001
Time to CTA (minutes)	0.986	0.973–1.000	0.046

OR, odds ratio; CI, confidence interval.

## 4 Discussion

### 4.1 Summary of findings

In this study, we evaluated the prognostic value of three commonly used EWS for predicting in-hospital mortality in patients with AAD. The overall in-hospital mortality rate was 33.3%. Univariable analysis demonstrated that higher EWS, older age, Type A AAD, GCS scores, elevated lactate and BUN lower bicarbonate levels, and the absence of surgical treatment were associated with an increased risk of in-hospital mortality. However, after multivariable adjustment, only Type A AAD and surgical treatment remained independent predictors of in-hospital mortality.

### 4.2 EWS and their predictive value

Among the three EWS evaluated, NEWS demonstrated the highest AUC, however, its overall discriminative performance was poor (AUC = 0.581). The AUCs of MEWS and SEWS were similarly low (0.57 and 0.56), indicating poor discriminative ability across all scores. Nevertheless, NEWS showed the highest AUC among the evaluated scores and was therefore selected for inclusion in the multivariable logistic regression model.

The relatively better performance of NEWS may be attributable to its incorporation of multiple physiological parameters, including respiratory rate, oxygen saturation, systolic blood pressure, heart rate, body temperature, and level of consciousness, which together provide a comprehensive assessment of acute physiological derangement (17). Compared with MEWS and SEWS, NEWS incorporates multiple physiological parameters that may explain its relatively higher AUC. However, NEWS was not independently associated with in-hospital mortality after adjustment for other clinical variables. This finding suggests that although physiological abnormalities assessed by NEWS are associated with mortality risk, their prognostic value may largely reflect underlying disease severity and clinical presentation rather than providing independent prognostic information. Consequently, NEWS should be considered a limited tool for early risk assessment rather than a standalone predictor of clinical outcomes in patients with AAD.

### 4.3 Age, AAD type, and laboratory markers as prognostic factors

Age was significantly associated with in-hospital mortality in the univariable analysis and remained an independent predictor among patients with Type A AAD. This finding is consistent with existing literature, which highlights advanced age as a risk factor for poor outcomes in acute cardiovascular conditions, including AAD (18–20). Older patients may have comorbidities or diminished physiological reserves, contributing to higher mortality risks.

Type A AAD was independently associated with increased in-hospital mortality in the overall multivariable analysis. Because Type A AAD involves the ascending aorta, it is frequently complicated by life-threatening conditions such as cardiac tamponade, acute aortic regurgitation, coronary malperfusion, and aortic rupture, all of which contribute to its substantially higher mortality compared with type B AAD (21). These findings further emphasize the importance of prompt diagnosis and timely surgical intervention in improving patient outcomes.

Although elevated lactate and BUN levels and lower bicarbonate levels were associated with increased in-hospital mortality in the univariable analysis, these associations were no longer statistically significant after multivariable adjustment. Recent evidence has also highlighted the prognostic value of inflammatory biomarkers in cardiovascular surgery. An observational study demonstrated that elevated intraoperative and postoperative Delta Neutrophil Index (DNI), a marker of circulating immature neutrophils, was independently associated with increased in-hospital mortality following cardiopulmonary bypass (22). Although this study involved a different patient population, it supports the concept that biomarkers reflecting systemic inflammatory responses may provide complementary prognostic information alongside physiological assessment, thereby improving early risk stratification in critically ill cardiovascular patients.

### 4.4 Subgroup analysis and type A vs. type B AAD

The subgroup analyses provided additional insight into the prognostic factors associated with different AAD subtypes. Age, surgical treatment, and time to CTA remained independently associated with in-hospital mortality among patients with Type A AAD, whereas NEWS and laboratory variables were no longer significant after multivariable adjustment. These findings suggest that definitive treatment and timely diagnosis may have a greater impact on clinical outcomes than physiological abnormalities alone in this high-risk population.

In contrast, among patients with type B AAD, surgical treatment was the only variable significantly associated with in-hospital mortality in the univariable analysis, while no independent prognostic factors were identified. This difference may reflect the distinct clinical characteristics and treatment strategies of type B AAD, which is more commonly managed conservatively and generally has a lower early mortality than Type A AAD. In addition, the relatively smaller sample size and lower number of mortality events in the type B subgroup may have limited the statistical power to detect independent associations.

### 4.5 Clinical implications

Our findings support that NEWS showed relatively better performance among the evaluated EWS during the initial assessment of patients with acute aortic dissection. Although its overall discriminatory performance was modest and it was not independently associated with in-hospital mortality after multivariable adjustment, NEWS may have limited value for early risk stratification in patients with acute aortic dissection.

More importantly, our findings highlight the critical importance of disease type and timely surgical treatment in determining clinical outcomes. Patients with suspected Type A AAD should undergo rapid diagnostic assessment and, when appropriate, urgent referral for definitive surgical management. Future prognostic models may achieve greater predictive accuracy by integrating EWS with disease-specific clinical characteristics, laboratory biomarkers, and imaging findings rather than relying on physiological scoring systems alone. These findings suggest that physiological EWS have limited standalone prognostic value and should be interpreted alongside disease-specific clinical assessment in patients with acute aortic dissection.

### 4.6 Limitations

This study has several limitations. First, this was a single-center retrospective study, and although rigorous statistical analyses were performed, selection bias, potential model overfitting, limited generalizability, and lack of external validation cannot be completely excluded. Second, 195 of 705 potentially eligible patients (27.7%) were excluded because of incomplete clinical data required for the calculation of EWS or key laboratory variables. Consequently, the study population may not be fully representative of all patients with acute aortic dissection, particularly those who were too critically ill to undergo complete assessment or those with incomplete documentation.

Third, although NEWS demonstrated the highest AUC among the evaluated EWS for predicting in-hospital mortality, its overall predictive performance remained modest. Therefore, these findings should not be interpreted as supporting the use of physiological EWS as standalone prognostic tools for patients with AAD. Incorporating disease-specific clinical characteristics, laboratory biomarkers, and imaging findings may improve predictive performance.

Finally, the findings of this study require external validation. Future prospective, multicenter studies with larger sample sizes are warranted to validate our results and to develop more comprehensive prognostic models for patients with acute aortic dissection.

## 5 Conclusion

NEWS demonstrated the highest discriminative ability among the evaluated EWS for predicting in-hospital mortality in patients with acute aortic dissection, although its overall predictive performance was modest. Type A AAD and surgical treatment were identified as the only independent predictors of in-hospital mortality in the overall cohort. These findings support the use of NEWS may have limited value for early risk stratification and should be interpreted together with other clinical indicators in patients with acute aortic dissection.

## Data Availability

The raw data supporting the conclusions of this article will be made available by the authors, without undue reservation.
